# M Segment-Based Minigenomes and Virus-Like Particle Assays as an Approach To Assess the Potential of Tick-Borne *Phlebovirus* Genome Reassortment

**DOI:** 10.1128/JVI.02068-18

**Published:** 2019-03-05

**Authors:** Veronica V. Rezelj, Timothy J. Mottram, Joseph Hughes, Richard M. Elliott, Alain Kohl, Benjamin Brennan

**Affiliations:** a MRC–University of Glasgow Centre for Virus Research, Glasgow, Scotland, United Kingdom; University of Southern California

**Keywords:** *Phlebovirus*, SFTS phlebovirus, minigenome, negative-strand RNA virus, reverse genetic analysis

## Abstract

Bunyaviruses have a tripartite negative-sense RNA genome. Due to the segmented nature of these viruses, if two closely related viruses coinfect the same host or vector cell, it is possible that RNA segments from either of the two parental viruses will be incorporated into progeny virions to give reassortant viruses. Little is known about the ability of tick-borne phleboviruses to reassort. The present study describes the development of minigenome assays for the tick-borne viruses Uukuniemi phlebovirus (UUKV) and Heartland phlebovirus (HRTV). We used these minigenome assays in conjunction with the existing minigenome system of severe fever with thrombocytopenia syndrome (SFTS) phlebovirus (SFTSV) to assess the abilities of viral N and L proteins to recognize, transcribe, and replicate the M segment-based minigenome of a heterologous virus. The highest minigenome activity was detected with the M segment-based minigenomes of cognate viruses. However, our findings indicate that several combinations utilizing N and L proteins of heterologous viruses resulted in M segment minigenome activity. This suggests that the M segment untranslated regions (UTRs) are recognized as functional promoters of transcription and replication by the N and L proteins of related viruses. Further, virus-like particle assays demonstrated that HRTV glycoproteins can package UUKV and SFTSV S and L segment-based minigenomes. Taken together, these results suggest that coinfection with these viruses could lead to the generation of viable reassortant progeny. Thus, the tools developed in this study could aid in understanding the role of genome reassortment in the evolution of these emerging pathogens in an experimental setting.

**IMPORTANCE** In recent years, there has been a large expansion in the number of emerging tick-borne viruses that are assigned to the *Phlebovirus* genus. Bunyaviruses have a tripartite segmented genome, and infection of the same host cell by two closely related bunyaviruses can, in theory, result in eight progeny viruses with different genome segment combinations. We used genome analogues expressing reporter genes to assess the abilities of *Phlebovirus* nucleocapsid protein and RNA-dependent RNA polymerase to recognize the untranslated region of a genome segment of a related phlebovirus, and we used virus-like particle assays to assess whether viral glycoproteins can package genome analogues of related phleboviruses. Our results provide strong evidence that these emerging pathogens could reassort their genomes if they were to meet in nature in an infected host or vector. This reassortment process could result in viruses with new pathogenic properties.

## Abstract (precis)

In recent years, there has been a large expansion in the number of emerging tick-borne viruses that are assigned to the *Phlebovirus* genus. Bunyaviruses have a tripartite segmented genome, and infection of the same host cell by two closely related bunyaviruses can, in theory, result in eight progeny viruses with different genome segment combinations. We used genome analogues expressing reporter genes to assess the abilities of *Phlebovirus* nucleocapsid protein and RNA-dependent RNA polymerase to recognize the untranslated region of a genome segment of a related phlebovirus, and we used virus-like particle assays to assess whether viral glycoproteins can package genome analogues of related phleboviruses. Our results provide strong evidence that these emerging pathogens could reassort their genomes if they were to meet in nature in an infected host or vector. This reassortment process could result in viruses with new pathogenic properties.

## INTRODUCTION

Tick-borne (TiBo) phleboviruses are a group of emerging viruses of the *Phlebovirus* genus within the *Phenuiviridae* family (order *Bunyavirales*) (1). Tick-borne phleboviruses have a trisegmented RNA genome composed of small (S), medium (M), and large (L) segments. A negative-sense coding strategy is utilized by the M and L segments. While the M segment encodes the glycoprotein precursor, which is cleaved for the generation of the surface glycoproteins Gn and Gc, the L segment encodes the viral RNA-dependent RNA polymerase (RdRp, or L protein). The S segment utilizes an ambisense coding strategy, by which the nucleocapsid protein (N) is translated from subgenomic mRNA transcribed from viral genomic RNA, and the nonstructural protein (NSs) is translated from subgenomic mRNA transcribed from antigenomic RNA (2).

Due to the segmented nature of these viruses, if two closely related viruses coinfect the same host or vector cell, RNA segments from either of the two parental viruses could be packaged into progeny virions. This genetic exchange phenomenon is known as genome reassortment and plays an important role in the evolution of segmented viruses (3).

Because bunyaviruses have a tripartite segmented genome, infection of the same host cell by two closely related bunyaviruses can, in theory, result in eight progeny viruses with different genome combinations: AAA, ABA, ABB, AAB, BBB, BAB, BBA, and BAA (where A and B refer to parental viruses with genomic segments S_A_, M_A_, and L_A_ and S_B_, M_B_, and L_B_, respectively). However, most recognized bunyavirus reassortants possess the S and L segments derived from one virus and the M segment from another virus (4). This has been suggested to be due to the intricate interplay required between the nucleocapsid protein (encoded by the S segment) and the viral polymerase (encoded by the L segment), which need to be functionally compatible for transcription and replication to occur (4). The acquisition of a new M segment can introduce radical phenotypic changes into the progeny reassortant viruses, such as new immunogenic characteristics and alterations in tropism, transmissibility, or host range (4, 5).

Perhaps the best-known occurrence of natural genome reassortment within the *Bunyavirales* is that of Ngari orthobunyavirus, which emerged in 1997 and caused a large outbreak of hemorrhagic fever in East Africa (6). This virus was later reported to be a reassortant virus, the progeny of two closely related orthobunyaviruses: Bunyamwera orthobunyavirus (BUNV) (S and L segment donor) and Batai orthobunyavirus (BATV) (M segment donor) (7). Intriguingly, 2 years before the outbreak, Dunn et al. had predicted that BUNV and BATV could reassort, a hypothesis based on experimental evidence showing that BUNV and BATV proteins were compatible for the transcription of RNA template minigenomes (8). Another bunyavirus, named Schmallenberg orthobunyavirus, emerged in 2012 and was reported to cause congenital defects in newborn calves, goats, and lambs (9). It was later found that Schmallenberg orthobunyavirus was a reassortant of two other viruses, namely, Sathuperi orthobunyavirus (M segment donor) and Shamonda orthobunyavirus (S and L segment donor) (10). Within the *Phlebovirus* genus, a genome analysis of Rift Valley fever phlebovirus (RVFV) indicated that reassortment events among RVFV strains had occurred over the evolutionary history of the lineage (11). Additionally, Aguacate phlebovirus has been suggested to be a natural reassortant from as yet unknown viruses (12), and Granada phlebovirus is likely a reassortant of Massilia phlebovirus (S and L segment donor) and a currently unidentified phlebovirus (M segment donor) (13). Recent efforts to characterize members of the *Phenuiviridae* have shown that several mosquito-borne members of this family are reassortants of existing or unidentified viruses (11–13).

Little is known about the ability of tick-borne phleboviruses to reassort. In addition, no experimental evidence exists to support the above-mentioned phylogenetic studies suggesting that certain members of the *Phenuiviridae* are reassortant progeny. The availability of reverse genetics systems could enable us to understand the potential of genome reassortment under experimental conditions by assessing the compatibility of viral proteins derived from two different viruses and their ability to form viable progeny. So far, reverse genetics systems for the tick-borne Uukuniemi phlebovirus (UUKV) (14, 15) and severe fever with thrombocytopenia syndrome phlebovirus (SFTSV) (16) have been described. Here we describe the development of a minigenome system for Heartland phlebovirus (HRTV), another tick-borne *Phlebovirus*, which is closely related to SFTSV. HRTV was originally isolated in the United States and was associated with two deaths (17, 18). Recent phylogenetic studies show that SFTSV and HRTV are closely related sister species in the SFTS group, while UUKV is more distantly related and is found in the Uukuniemi group, another known lineage of tick-borne phleboviruses (19). Using a combination of minigenome and virus-like particle (VLP) assays, we investigated the abilities of tick-borne *Phlebovirus* proteins to replicate, transcribe, and package the genomes of related viruses. These tools could allow us to understand whether genome reassortment acts as a driving force behind the evolution of these emerging viruses and to predict, in an experimental setting, the possible outcomes of reassortant viruses in nature. In the absence of functional reverse genetics systems for all the viruses described here, such studies could allow for the prediction of potential emerging reassortant tick-borne phleboviruses and, as such, influence the timely development of vaccines.

## RESULTS

### Development of an HRTV minigenome system.

Minigenome systems for bunyaviruses commonly consist of the expression of viral N and L proteins along with a minigenome plasmid carrying a reporter gene flanked by the viral untranslated regions (UTRs) in the viral genomic sense under the control of a T7 RNA polymerase (RNAP) promoter, hepatitis δ virus ribozyme (Hδr), and T7 terminator sequence. In the presence of T7 RNAP, expressed N and L proteins can encapsidate the negative-sense reporter genome analogue generated by T7 RNAP to generate artificial RNPs and can utilize the viral UTRs as promoters of replication and transcription to drive the expression of the reporter gene.

We sought to develop S, M, and L segment-based minigenome systems for HRTV similar to the SFTSV S and M segment-based minigenomes described previously (16). First, HRTV S, M, and L segment-based minigenome plasmids were created and were named pT7HRTSRen(+), pT7HRTMRen(–), and pT7HRTLRen(–), respectively. Of note, the pT7HRTMRen(–) and pT7HRTLRen(–) constructs contain the humanized *Renilla* (Ren) luciferase open reading frame (ORF) sequence in the negative sense, flanked by the viral UTR in the genomic sense. On the other hand, pT7HRTSRen(+) contains the *Renilla* luciferase ORF sequence in the negative sense in place of the NSs ORF. Helper plasmids expressing HRTV N and L proteins in the T7 RNAP-driven expression vector pTM1 (20) were also created and are called pTM1HRTN and pTM1HRTL.

To determine whether the minigenome constructs would allow the generation of functional RNPs that are transcription and replication competent, a minigenome assay was carried out. Briefly, subconfluent monolayers of cells of the BSR-T7/5 line (a BHK-21 cell-derived clone that constitutively expresses T7 RNAP) (21) were transfected with constant amounts of pT7HRTSRen(+), pT7HRTMRen(–), or pT7HRTLRen(–), as well as pTM1HRTN and a plasmid expressing firefly luciferase (pTM1FFluc) as an internal control for transfection efficiency, and increasing amounts (0 to 375 ng) of pTM1HRTL. The total amount of DNA per well was kept constant by supplementing the DNA reaction mixtures with empty pTM1. At 18 h posttransfection, the cells were harvested, and *Renilla* and firefly luciferase activities were measured. *Renilla* luciferase values were normalized to firefly luciferase values, and minigenome activity was expressed as the fold increase in normalized luciferase units over the level for the background control (in the absence [0 ng] of L protein).

**FIG 1:**
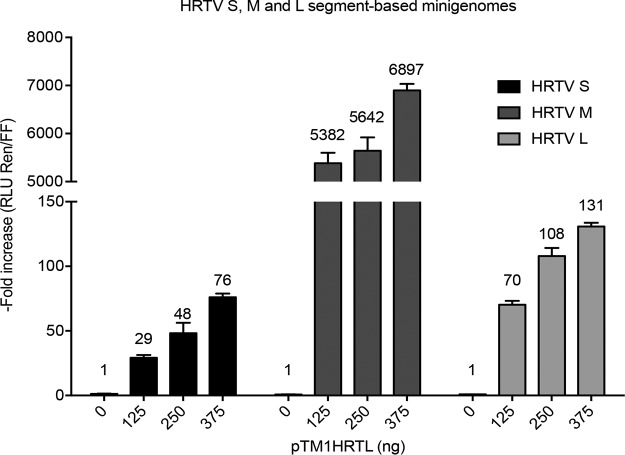
Development of HRTV S, M, and L minigenome assays. BSR-T7/5 cells were transfected with constant amounts of pT7HRTSRen(+) (filled bars), pT7HRTMRen(–) (dark shaded bars), or pT7HRTLRen(–) (light shaded bars), as well as pTM1HRTN and pTM1FFluc, and increasing amounts (0 to 375 ng) of pTM1HRTL. The total amount of plasmid DNA in each reaction mixture was kept constant by the addition of the empty control plasmid pTM1 to the DNA mixture. Cells were lysed 18 h posttransfection, and the *Renilla* and firefly luciferase units were measured. Minigenome activity is expressed as the fold induction of normalized luciferase units relative to activity for the background control (no pTM1HRTL). The mean minigenome activities calculated are shown above the bars. Each bar represents the mean fold induction for three experimental repeats; error bars, SD.

Collectively, all minigenome constructs resulted in a dose-dependent increase in normalized luciferase activity when pTM1HRTL was provided (Fig. 1). The highest fold increase in normalized luciferase activity was observed for the HRTV M segment-based minigenome, which resulted in a mean 6,897-fold increase over the level for the background control when 375 ng of pTM1HRTL was supplied. This is in contrast to the S and L segment-based minigenomes, which yielded mean peak 76- and 131-fold increases in luciferase activities, respectively (Fig. 1). These results confirmed that the cDNA clones of the N and L proteins, as well as the viral UTRs belonging to the three segments in the published HRTV sequences, are functional and enable replication and transcription processes to occur.

### Assessing the abilities of UUKV, HRTV, and SFTSV N and L proteins to utilize the M segment minigenomes of heterologous tick-borne phleboviruses.

The abilities of N and L proteins to recognize the viral UTRs as promoters of transcription and replication will determine the functionality of a minigenome. Compatible viral components (such as N and L proteins of a given *Phlebovirus* that can recognize the viral UTRs of a segment belonging to a heterologous *Phlebovirus*) are a key requirement for reassortment between closely related viruses, such as these tick-borne phleboviruses. Therefore, the availability of functional tick-borne *Phlebovirus* minigenome systems was exploited to investigate whether the N and L proteins of one virus could transcribe and replicate the M segment genome analogues of other tick-borne phleboviruses. For this study, the activity of the M segment-based minigenome using N and L proteins belonging to a different virus was considered an indicator of potential for reassortment between the viruses tested. The tick-borne *Phlebovirus* M segment-based minigenome systems currently available include those of UUKV (22), HRTV (the present study), and SFTSV (16). Of note, because the currently available UUKV minigenome system is driven by RNAP I (22), T7 RNAP-driven plasmids such as those described for HRTV were generated for UUKV, namely, pT7UUKSRen(+), pT7UUKMRen(–), pT7UUKLRen(–), pTM1UUKN, and pTM1UUKL. The SFTSV M segment-based minigenome construct (pTVT7HB29M:hRen) and the SFTSV N- and L-expressing plasmids pTM1-HB29N and pTM1-HB29L, respectively, have been described previously (16).

The abilities of the N and L proteins of UUKV, HRTV, and SFTSV to replicate and transcribe M segment-based minigenomes belonging to heterologous viruses were tested, using the minigenomes of cognate viruses as controls. The M segment-based minigenome system was chosen because, as mentioned above, reassortant viruses with a novel M segment are of major concern. In addition, most bunyavirus reassortants identified to date possess S and L segments derived from one virus and an M segment derived from another virus, indicating that reassortment may be restricted to the M segment (4). Assays were carried out utilizing N and L proteins from cognate viruses only, and the abilities of N and L proteins from different viruses to function together and to recognize, replicate, and transcribe a given minigenome were not tested.

**FIG 2:**
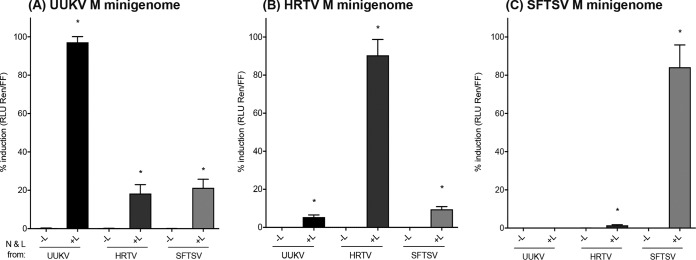
Activities of the UUKV, HRTV, and SFTSV M segment-based minigenomes with UUKV, HRTV, or SFTSV N and L proteins. BSR-T7/5 cells were transfected with pT7UUKMRen(–) (A), pT7HRTMRen(–) (B), or pTVT7HB29M:hRen (C), together with pTM1FFluc, pTM1XN, and pTM1XL (where X refers to UUKV, HRTV, or SFTSV). Cells were lysed 18 h posttransfection and the *Renilla* and firefly luciferase units measured. Minigenome activity is expressed as the fold induction of normalized luciferase units relative to the background control (absence of L-expressing plasmid), corrected as a percentage of the activity observed in the presence of homologous N and L proteins for each minigenome. Each bar represents the mean percentage of the fold induction for three experimental repeats (*, *P* < 0.05). Error bars, SD.

BSR-T7/5 cells were transfected with constant amounts of one of the M segment-based minigenomes [pT7UUKMRen(–) for UUKV, pT7HRTMRen(–) for HRTV, or pTVT7HB29M:hRen for SFTSV (strain HB29)] and the firefly luciferase expression plasmid pTM1FFluc and with 125 ng of N- and L-expressing plasmids (pTM1XN and pTM1XL, where X stands for UUKV, HRTV, or SFTSV [strain HB29]). Control wells lacked L-expressing plasmids, and the DNA reaction mixtures were supplemented with empty pTM1 to keep the total amount of DNA constant under all conditions. At 18 h posttransfection, the cells were harvested, and *Renilla* and firefly luciferase values were determined. *Renilla* luciferase values were normalized to firefly luciferase values, and minigenome activity was expressed as the normalized luciferase activity relative to the background control (absence of L-expressing plasmid [–L]). As expected, the M segment-based minigenomes of UUKV, HRTV, and SFTSV exhibited the highest activity when each minigenome system was supplied with cognate N and L proteins (Fig. 2A to C). Interestingly, the HRTV and SFTSV N and L proteins could recognize the UUKV M segment UTRs as a promoter for transcription and replication, resulting in a mean rescue of minigenome activity equivalent to 18.3% and 21.3%, respectively, of the UUKV M minigenome activity observed when UUKV N and L proteins were used (Fig. 2A). The UUKV and SFTSV N and L proteins were also able to promote HRTV M minigenome activity, resulting in 5.4% and 9.5% mean rescue of minigenome activity, respectively (Fig. 2B). Finally, only HRTV N and L (but not UUKV N and L) proteins promoted SFTSV M minigenome activity, yielding only 1.4% of the minigenome activity observed when SFTSV N and L proteins were used (Fig. 2C). Taken together, these results demonstrate that the UUKV N and L proteins can recognize and utilize the HRTV M UTRs but not the SFTSV M UTRs as a promoter, whereas the HRTV N and L proteins can recognize both UUKV and SFTSV M UTRs, and the SFTSV N and L proteins can recognize UUKV and HRTV M UTRs, for the transcription and replication of M segment-based minigenomes (summarized in Table 1).

**TABLE 1:** Summary of UUKV, HRTV, and SFTSV M segment-based minigenome activities detected in the presence of N and L proteins from cognate or heterologous viruses

Minigenome	Activity (% increase)^*a*^ in the presence of N and L proteins		
	UUKV	HRTV	SFTSV
UUKV	+ (97.1)	+ (18.3)	+ (21.3)
HRTV	+ (5.4)	+ (90.4)	+ (9.5)
SFTSV	–	+ (1.4)	+ (85)

a +, increase in minigenome activity for the indicated M segment-based minigenome in the presence of the indicated N and L proteins; –, no increase in minigenome activity. The percentages of minigenome activity increase given in parentheses are relative to the activity of the background control in the absence of L protein. One hundred percent activity is defined as the maximum increase in activity recorded using N and L proteins of the cognate virus.

### HRTV GnGc can package UUKV and SFTSV S and L segment-based minigenomes.

The abilities of the N and L proteins of a given virus to recognize, transcribe, and replicate the M segment of a heterologous virus are a prerequisite for reassortment between two coinfecting viruses. Moreover, other factors, such as the ability of the M segment products (i.e., the viral glycoproteins) to package the S and L segments of heterologous viruses and produce viable progeny, must also be considered. To address this question, we carried out virus-like particle (VLP) assays using S or L segment-based UUKV, HRTV, or SFTSV minigenomes in the presence of plasmids expressing the UUKV, HRTV, or SFTSV glycoproteins Gn and Gc. Briefly, Huh7-Lunet-T7 cells (which were used due to a decreased infection efficiency of SFTSV in BHK-derived cell lines [data not shown]) were transfected with the S or L segment-based *Renilla* luciferase minigenome-carrying plasmids of UUKV, HRTV, or SFTSV, plasmids encoding the corresponding N and L proteins, and a firefly luciferase-expressing plasmid as a transfection control. The DNA mixture was supplemented with plasmids carrying and expressing the glycoprotein (M) ORF of UUKV, HRTV, or SFTSV (pTM1XGnGc, where X stands for UUKV, HRTV, or SFTSV). At 72 h posttransfection, the cells were assayed for a luciferase signal (see Fig. S2 in the supplemental material), and the cell culture medium of donor cells was clarified by centrifugation and treated with nuclease. The nuclease-treated supernatant containing VLPs was used to inoculate Huh7-Lunet-T7 cells transiently expressing UUKV, HRTV, or SFTSV N and L proteins (homologous to the nature of the minigenome investigated). *Renilla* and firefly luciferase activities were measured in the lysates of infected recipient cells.

**FIG 3:**
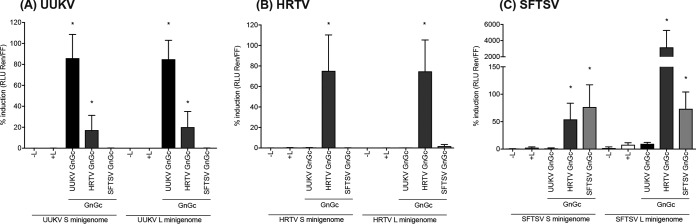
Activities of virus-like particles containing S or L segment-based minigenomes with UUKV, HRTV, or SFTSV glycoproteins. VLPs containing UUKV (A), HRTV (B), or SFTSV (C) S or L segment-based minigenomes were generated in Huh7-Lunet-T7 donor cells, using UUKV, HRTV, or SFTSV glycoproteins. Mock controls included a lack of L protein (–L) or a lack of glycoprotein-coding plasmids (+L) in the transfection reaction mixtures. Treated lysates from donor cells were used to inoculate transiently transfected Huh7-Lunet-T7 cells. At 24 h p.i., *Renilla* and firefly luciferase activities in infected-cell lysates were measured. VLP activity is expressed as the fold induction of normalized luciferase units relative to the background control (absence of a GnGc-expressing plasmid), corrected as a percentage of the activity observed in the presence of homologous GnGc for each VLP. Each bar represents the mean percentage of the fold induction for four experimental repeats (*, *P* < 0.05). Error bars, SD.

In the presence of all glycoproteins, minigenome activity could be detected in donor cells (Fig. S2 in the supplemental material). Figure 3 shows that, as expected, UUKV S and L segment-based minigenomes were efficiently packaged by UUKV glycoproteins, with mean 365- and 1,142-fold increases in luciferase activity detected in recipient cells infected with VLPs generated using UUKV GnGc over the level in the control VLP assay where no L protein was added to donor cells (Fig. 3A). Although no increase in UUKV S and L segment-based reporter minigenome activity was observed in recipient cells infected with VLPs derived from SFTSV GnGc-expressing donor cells, 17.3% and 20.1% of reporter activity were recovered with VLPs derived from HRTV GnGc-expressing donor cells. These results suggested that HRTV GnGc could package UUKV S and L segment-based minigenomes.

VLP assays using the HRTV S segment-based minigenome as a reporter showed that only HRTV GnGc, but not UUKV or SFTSV GnGc, could package the HRTV S minigenome. VLPs containing the HRTV L minigenome and generated using HRTV glycoproteins yielded a mean 1,057-fold increase in luciferase activity in recipient cells over that with the mock VLPs (generated in donor cells containing no L protein or GnGc). When HRTV L-containing VLPs were generated using SFTSV GnGc, 1.85% of this activity was recovered, whereas no activity was detected in the presence of UUKV GnGc (Fig. 3B).

VLPs packaging the SFTSV S segment-based minigenome and generated with SFTSV glycoproteins yielded a mean 95-fold increase in luciferase activity in recipient cells, whereas those generated using HRTV glycoproteins yielded a mean 60-fold increase. Interestingly, while VLPs packaging the SFTSV L segment-based minigenome and generated with SFTSV glycoproteins yielded a mean 24-fold increase in luciferase activity in recipient cells, those generated using HRTV glycoproteins yielded a mean 3,174% increase in activity, suggesting more efficient packaging of the SFTSV L segment by HRTV GnGc than by SFTSV GnGc. A summary of these results is shown in Table 2.

**TABLE 2:** Summary of S and L segment-based VLPs using glycoproteins from cognate or heterologous viruses

Minigenome	Activity (% increase)^*a*^ in the presence of glycoproteins from:		
	UUKV	HRTV	SFTSV
UUKV S	+ (85.9)	+ (17.3)	–
UUKV L	+ (85)	+ (20.1)	–
HRTV S	–	+ (75)	–
HRTV L	–	+ (75)	+ (1.85)
SFTSV S	–	+ (54.2)	+ (76.6)
SFTSV L	–	+ (3,173.9)	+ (73.5)

a +, increase in minigenome activity in recipient cells infected with VLPs generated in the presence of the indicated GnGc proteins; –, no increase in minigenome activity in recipient cells infected with VLPs. The percentages of minigenome activity increase given in parentheses are relative to the activity of the background control in the absence of L protein. One hundred percent activity is defined as the maximum increase in activity recorded using N and L proteins of the cognate virus.

### Altering the minigenome UTR structure affects activity using a heterologous nucleocapsid protein and RNA-dependent RNA polymerase.

**FIG 4:**
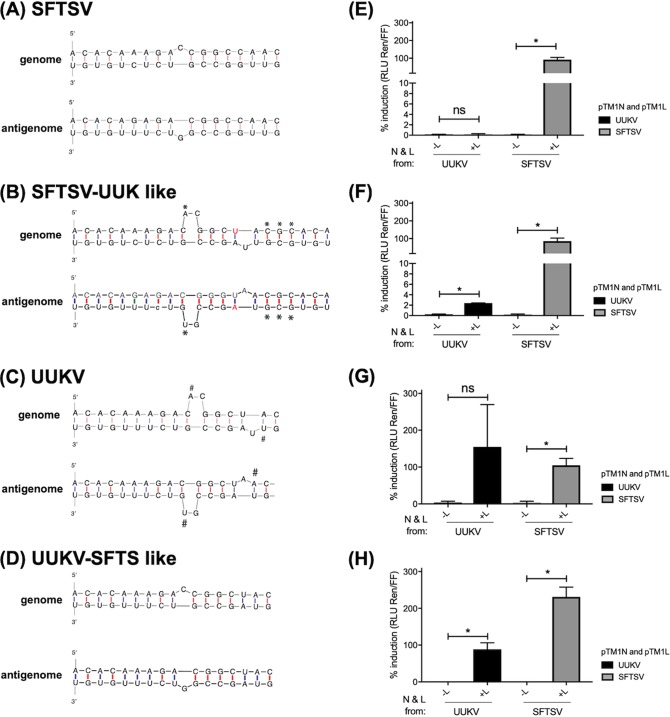
Activities of wild-type or mutated UUKV or SFTSV M segment-based minigenomes. (A through D) Genomic and antigenomic UTR structures were predicted by the Mfold Web server for SFTSV, SFTSV-UUK like, UUKV, and UUKV-SFTS like. To generate the “like” plasmids, nucleotides in the UTR sequence were replaced (red), added (*), or deleted (#). BSR-T7/5 cells were transfected with cDNA plasmids expressing a genomic sense RNA for pTVT7HB29M:hRen (A), pTVT7HB29M:hRen-UUKlike (B), pT7UUKMRen(–) (C), or pT7UUKMRen(–)SFTSlike (D), pTM1FFluc, pTM1XN, and pTM1XL (where X stands for UUKV or SFTSV). (E through H) Cells were lysed 18 h posttransfection, and *Renilla* and firefly luciferase units were determined. The minigenome activities of SFTSV (E), SFTSV-UUK like (F), UUKV (G), and UUK-SFTS like (H) are expressed as the fold induction of normalized luciferase units relative to the background control (absence of an L-expressing plasmid), corrected as a percentage of the activity observed in the presence of homologous N and L proteins for each minigenome. Each bar represents the mean percentage of the fold induction for three experimental repeats (*, *P* < 0.05). Error bars, SD.

It was noted from our data that the UUKV M minigenome was poorly recognized by the SFTSV nucleocapsid protein and viral RNA-dependent RNA polymerase (Fig. 2A) and that both the SFTSV and HRTV M minigenomes were very poorly recognized by the UUKV N and L proteins (Fig. 2B and C). It is known that the nucleotide sequences of the viral UTRs and the RNA structures that they form allow the recognition and binding of viral proteins to form the viral RNPs. To examine whether nucleotide substitution, and hence UTR RNA structure, impacted the heterologous recognition of N and L proteins from a related *Phlebovirus*, the SFTSV M minigenome was made UUKV-like, and vice versa, through RNA structural analysis and nucleotide substitutions (Fig. 4) We found no structural difference between the HRTV and SFTSV UTRs in the regions examined; therefore, only SFTSV UTRs were tested (the antigenomic HRTV UTR structure is shown in Fig. S3 in the supplemental material). When the SFTSV M minigenome was made UUKV-like, there was a small but reproducible 5-fold increase in activity using the UUKV N and L proteins (Fig. 4E and F). Making the UUKV M minigenome more SFTSV-like resulted in twice as much luciferase activity when the minigenome was assayed with the SFTSV N and L proteins (Fig. 4G and H). These data suggest that even small changes to the UTR sequence, such as those that could occur through selection pressure from mammalian hosts or arthropod vector cellular environments, could facilitate reassortment through the replication and transcription of viral RNAs by heterologous phleboviral proteins.

## DISCUSSION

As tick-borne phleboviruses continue to emerge in new geographical locations with increasing host ranges (5), there is an urgent need to understand the viral determinants that contribute to the evolution of these viruses and their escape from immune surveillance. Genome reassortment has long been acknowledged as a key force driving the evolution of the *Bunyavirales* (23) and other segmented viruses. In fact, Briese et al. hypothesized that all known bunyaviruses may represent reassortants of existing or extinct viruses (4). This hypothesis emphasizes the need for reverse genetics systems for emerging bunyaviruses, since such tools allow the assessment of the potential for genome reassortment under experimental conditions.

Here we describe the development of a minigenome system for HRTV (Fig. 1). The developed HRTV minigenome system will serve as a useful tool to facilitate functional studies of the HRTV UTR sequences, N and L proteins, and other transcriptional and replication processes. The UUKV, HRTV, and SFTSV minigenome systems were utilized as a first step to assess whether the N and L proteins of UUKV, HRTV, or SFTSV could recognize, transcribe, and replicate the M segment-based minigenome of each of these tick-borne phleboviruses. A negative-sense reporter construct was used to ensure the minimum background expression of luciferase activity in the system. Positive-sense reporter constructs can give false readings due to leaky ribosomal translation of the reporter gene. As expected, the highest minigenome activity was detected when N and L proteins were used in combination with the M segment-based minigenome of the respective virus, demonstrating that utilizing N and L proteins and the M segment UTRs from cognate viruses results in optimal minigenome activity (Fig. 2). Interestingly, HRTV and SFTSV N and L proteins could utilize UUKV M UTRs as a functional promoter (Fig. 2A). Additionally, the results from this work revealed that UUKV N and L proteins recognized HRTV M UTRs, but not SFTSV M UTRs, as a functional promoter for transcription and replication (Fig. 2B and C, respectively). Finally, the N and L proteins of the closely related viruses HRTV and SFTSV recognized each other’s M segment UTRs as functional promoters and resulted in activity higher than that with the M RNA from UUKV, probably due to the closer phylogenetic relationship between SFTSV and HRTV than between either one and UUKV (Fig. 2B and C).

The abilities of the N and L proteins of a given phlebovirus to recognize the M segment UTRs of a heterologous *Phlebovirus* for subsequent transcription and replication indicate that, provided that the viral glycoproteins can package the S and L segments of a heterologous virus, a new reassortant virus with the S and L segments of the parent virus and the M segment of the heterologous virus could be generated upon coinfection of the same host cell. It should also be noted that in addition to recognition of the UTR structure, the percentage of activity will depend on recognition by the viral glycoproteins and the intrinsic transcription and replication activity rates of the nucleocapsid protein and RNA-dependent RNA polymerase encoded by each virus. Thus, the findings obtained in our minigenome assays indicate that of the six possible reassortment outcomes examined (**U**M/**H**S/**H**L, **U**M/**S**S/**S**L, **H**M/**U**S/**U**L, **H**M/**S**S/**S**L, **S**M/**U**S/**U**L, and **S**M/**H**S/**H**L [where the first letter stands for **U**UKV, **H**RTV, or **S**FTSV, and the second letter stands for the S, M, or L segment]), five reassortment combinations (**U**M/**H**S/**H**L, **U**M/**S**S/**S**L, **H**M/**U**S/**U**L, **H**M/**S**S/**S**L, and **S**M/**H**S/**H**L) are possible among the tick-borne phleboviruses tested.

Furthermore, our VLP assays demonstrate that UUKV S and L segment-based minigenomes, as well as SFTSV S and L segment-based minigenomes, can be packaged by HRTV glycoproteins (Fig. 3A and C). Thus, given that the N and L proteins of UUKV and SFTSV can recognize, transcribe, and replicate the M segment minigenome of HRTV, and the HRTV glycoproteins can package S and L segment minigenomes into VLPs, it is possible that if UUKV and HRTV or SFTSV and HRTV coinfect the same cell, viable reassortant progeny could be produced, comprising the M segment of HRTV and the S and L segments of UUKV or SFTSV, respectively. Of note, although SFTSV glycoproteins could package the HRTV L segment minigenome, no packaging of the HRTV S segment could be detected by VLP assays (Fig. 3B). Thus, it appears unlikely that progeny virions comprising the S and L segments of HRTV and the M segment of SFTSV can be generated. The increased activity seen in Fig. 3C when the SFTSV L minigenome was packaged by the HRTV glycoproteins was a surprising result. Of note, the ability of HRTV glycoproteins to package S and L minigenomes of both UUKV and SFTSV could indicate that these proteins have reduced specificity for the packaging of *Phlebovirus* segments.

A recent publication has described the potential for reassortment between several members of the *Peribunyaviridae* family (24). In that study, the authors related mismatches in the UTR sequences to the recognition of heterologous N and L proteins and minigenome functionality, through mutational analysis. We performed a similar analysis with UUKV or SFTSV UTRs as a proof of concept (Fig. 4). We found that by mutating viral UTRs from one M minigenome to mimic the predicted RNA structures of a different *Phlebovirus*, we could increase the recognition of the minigenome RNA by the nucleocapsid protein and RNA-dependent RNA polymerase of the second virus. This shows us that, should these viruses reassort or should mutations arise in the UTRs, these mutations could favor the generation and adaptation of heterologous viral proteins to the genomic RNA segments of another virus during a reassortment event. Further work detailing a greater range of nucleotide substitutions will be conducted to assess this in the future.

To conclude, our study provides the first experimental evidence for the possibility of reassortment between tick-borne phleboviruses. It must be noted that factors such as geographic and ecological conditions should also be considered in assessing the possibility of reassortment between tick-borne phleboviruses. For instance, the two potential donor viruses must be circulating within close proximity to each other in order to infect the same vector or host cell. Although it appears that the distributions of the tick-borne phleboviruses analyzed in this study are distinct (UUKV in northern Europe, HRTV in the United States, and SFTSV in China, Japan, and South Korea), the geographic distribution of tick-borne phleboviruses in general is global (25). Furthermore, climate change may induce alterations in the distributions of the vector tick species of these viruses, or in the flight routes of migratory birds known to carry ticks (26). Donor viruses must also be able to infect the same vector or host species. While UUKV has been isolated from Ixodes ricinus tick species (27), the primary tick vectors of HRTV and SFTSV are believed to be Amblyomma americanum (Lone Star tick) (28) and Haemaphysalis longicornis (29, 30), respectively. However, to our knowledge, the vector competence for these tick-borne phleboviruses has not been examined experimentally, and it is possible that these viruses can infect the same tick, even if it is not the primary vector. Recent experimental evidence has demonstrated the transstadial and transovarial transmission of SFTSV (31). Thus, an infected tick would only need to feed on one host infected with a different tick-borne virus to introduce two tick-borne phleboviruses into the same vector. In addition, the vertebrate hosts of these viruses remain unclear. Of note, the assays described in this article were performed in an experimental setting, and due to biosafety concerns, no coinfection work was carried out. However, it must be mentioned that important limitations exist for the emergence of possible new reassortant viruses, such as superinfection exclusion, transmission efficiency, and the greater fitness of fast-replicating viruses than of newly emergent, slow-replicating viruses, which require adaptation. Further evolutionary studies are necessary to understand the fitness and transmission of such reassortant viruses and their potential to emerge in nature. Although several serological surveys that examined the distributions of UUKV-, HRTV-, or SFTSV- specific antibodies in a number of different species have been reported (32–40), there is a need for further surveillance of animal and human populations in order to assess the risks of genome reassortment. Our minigenome and virus-like particle assays showed that certain viral components required for transcription, replication, and packaging are compatible between tick-borne phleboviruses. Our assays also highlighted potential reassortant viruses comprising the M segment of HRTV and the S and L segments of UUKV or SFTSV. However, further studies are required, once a functional HRTV reverse genetics system has been described, to confirm the ability of these viruses to reassort and produce viable progeny. Efforts to rescue reassortant viruses using reverse genetics under the appropriate biosafety conditions to confirm the results obtained in this study could help in predicting the possible outcomes of natural reassortant viruses, as has been demonstrated previously for the emergence of Ngari virus (8), and could allow the timely development of vaccines for those emerging viruses that can escape immune surveillance.

## MATERIALS AND METHODS

### Cells.

BSR-T7/5 cells stably expressing bacteriophage T7 RNA polymerase (T7 RNAP) (21) were maintained in Glasgow minimal essential medium (GMEM) supplemented with 10% fetal calf serum (FCS) and 10% tryptose phosphate broth under G418 selection. Huh7-Lunet-T7 cells (41) also stably expressing T7 RNAP were kindly provided by R. Bartenschlager (Universitätsklinikum, Heidelberg, Germany) and were maintained in Dulbecco’s modified Eagle’s medium (DMEM) supplemented with 10% FCS, 2 mM l-glutamine, and nonessential amino acids under Zeocin (250 μg/ml) selection. The cells were cultured at 37°C under 5% CO_2_.

### Plasmids and cloning.

Minigenome plasmids were generated similarly to those created previously for RVFV (42), using In-Fusion HD restriction-free cloning (Clontech). M and L segment-based minigenome constructs contain the humanized *Renilla* (hRen) ORF in the negative sense, flanked by genomic sense viral untranslated-region (UTR) sequences in the plasmid backbone TVT7R(0,0), which contains T7 RNAP promoter, T7 RNAP terminator, and hepatitis δ virus ribozyme sequences. S segment-based minigenome constructs contain the full-length antigenome-sense S segment of the virus in plasmid TVT7R(0,0), with the NSs ORF replaced by that of hRen. The plasmids were named pT7UUKSRen(+), pT7UUKMRen(–), and pT7UUKLRen(–) for UUKV minigenomes; pT7HRTSRen(+), pT7HRTMRen(–), and pT7HRTLRen(–) for HRTV minigenomes; and pT7SFTSLRen(–) for the SFTSV L segment-based minigenome. Details of the minigenome configurations used in this study can be found in Fig. S1 in the supplemental material.

Expression plasmids were based on the pTM1 clone (20), in which viral ORFs are under the control of a T7 RNAP promoter and an encephalomyocarditis virus internal ribosome entry site sequence, allowing protein expression by cap-independent translation (43). Plasmids pTM1UUKN, pTM1UUKL, pTM1HRTN, and pTM1HRTL encode the UUKV or HRTV nucleocapsid (N) or viral RNA polymerase (L). pTM1UUKGnGc, pTM1HRTGnGc, and pTM1SFTSGnGc encode the UUKV, HRTV, or SFTSV glycoprotein precursor, respectively. The plasmids were generated based on the following GenBank accession numbers: M33551.1 (UUKV S), M17417.1 (UUKV M), D10759.1 (UUKV L), JX005843.1 (HRTV S), JX005845.1 (HRTV M), JX005847.1 (HRTV L), KP202164.1 (SFTSV M), and KP202163 (SFTSV L). The SFTSV T7-driven N and L expression plasmids pTM1-HB29N and pTM1-HB29L and the minigenome plasmids pTVT7-HB29SdelNSs:hRen and pTVT7HB29M:hRen have been described previously (16).

### Minigenome assays.

HRTV minigenome assays were carried out by transfecting subconfluent BSR-T7/5 cells in 24-well plates with 25 ng pTMFFluc (to measure firefly luciferase activity as an internal control of transfection efficiency and data normalization), 125 ng pT7HRTSRen(+), pT7HRTMRen(–), or pT7HRTLRen(–), 250 ng pTMHRTN, and increasing amounts (0 to 375 ng) of pTMHRTL. The total amount of DNA in all reaction mixtures was kept constant by the addition of empty control plasmid pTM1 to the DNA mixtures. At 24 h posttransfection, the cell monolayer was washed with phosphate-buffered saline (PBS), lysed and used to measure firefly and *Renilla* luciferase activities by use of the Dual Luciferase Reporter Assay system (Promega) according to the manufacturer’s instructions.

### VLP assays.

The ability of S- or L-segment based minigenomes to be packaged into virus-like particles (VLPs) was tested using VLP assays, as described previously (44). Briefly, subconfluent Huh7-Lunet-T7 cells were transfected with 250 ng of the S or L segment-based minigenome-carrying plasmid (pT7XSRen or pT7XLRen), pTM1-XN, pTM1-XL, pTM1XGnGc (where X refers to UUKV, HRTV, or SFTSV), and pTM1-FF-Luc as a transfection control. At 72 h posttransfection, the cell culture medium was clarified by centrifugation at 10,000 × *g* for 10 min and was treated with 0.25 U/μl BaseMuncher (Expedeon) for 3 h at 37°C. Huh7-Lunet-T7 cells transiently expressing UUKV, HRTV, or SFTSV N and L proteins were inoculated with the VLP-containing cell culture medium. Twenty-four hours postinfection (p.i.), the infected cell lysates were used to measure *Renilla* and firefly luciferase activities by use of the Dual Luciferase Reporter Assay system (Promega).

### Predicted RNA structure analysis of phlebovirus M segment untranslated regions.

*In silico* RNA secondary-structure prediction was conducted using the Mfold Web server (45) with default settings at 37°C to determine the genomic and antigenomic panhandle structures of the M segment in UUKV (GenBank accession number M17417.1) and SFTSV (GenBank accession number KP202164.1). Based on the modeling, nucleotide additions, substitutions, or deletions were made to cDNA plasmids carrying SFTSV or UUKV M segment-based minigenomes. pTVT7HB29M:hRen-UUK like or pT7UUKMRen(–)SFTS like was generated by site-directed mutagenesis, and all cDNA constructs were confirmed by Sanger sequencing.

### Statistical analysis.

All data were analyzed using Prism 7 software (GraphPad) and are presented as means ± standard deviations (SD). Statistical significance for the comparison of means between two groups was determined by Student’s *t* test. *P* values of <0.05 were considered significant (*, *P* < 0.05).

## Supplementary Material

Supplemental file 1
