# Supplementary material for: M Segment-Based Minigenomes and Virus-Like Particle Assays as an Approach To Assess the Potential of Tick-Borne Phlebovirus Genome Reassortment
Source: J Virol. 2019 Mar 5;93(6):e02068-18. doi: 10.1128/JVI.02068-18 (PMC6401446; doi:10.1128/JVI.02068-18)
Supplement: Supplemental file 1 [file JVI.02068-18-s0001.pdf]

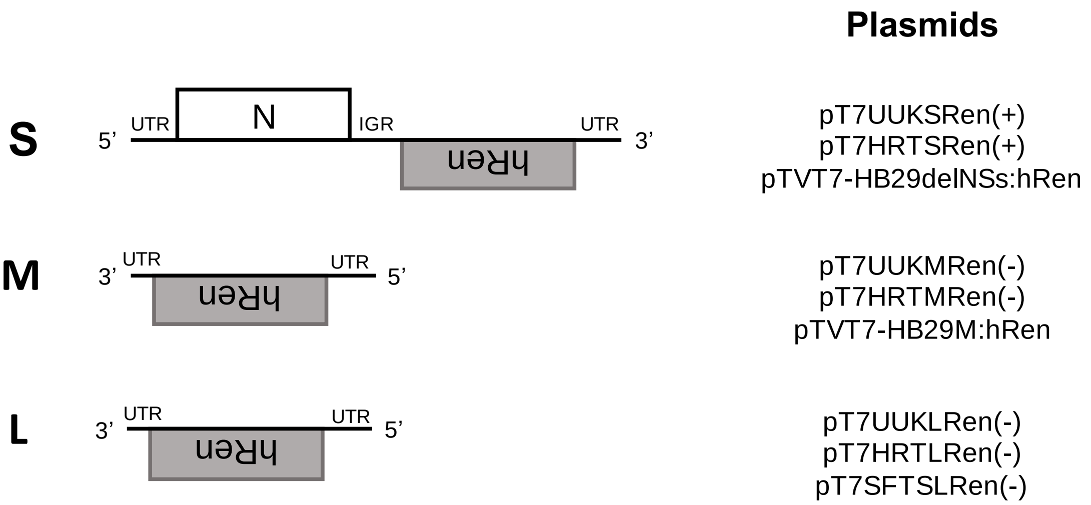

Fig S1. Minigenome configurations. A schematic diagram of the S-, M- and L-segment based humanised *Renilla* luciferease (hRen) containing minigenomes and the names of the plasmids which contain them. Orientation reflects the configuration of the RNA segment transcribed from the minigenome expressing plasmid. Segments are not drawn to scale. UUK: Uukuniemi phlebovirus; HRT: Heartland phlebovirus and HB29 or SFTS: SFTS phlebovirus.

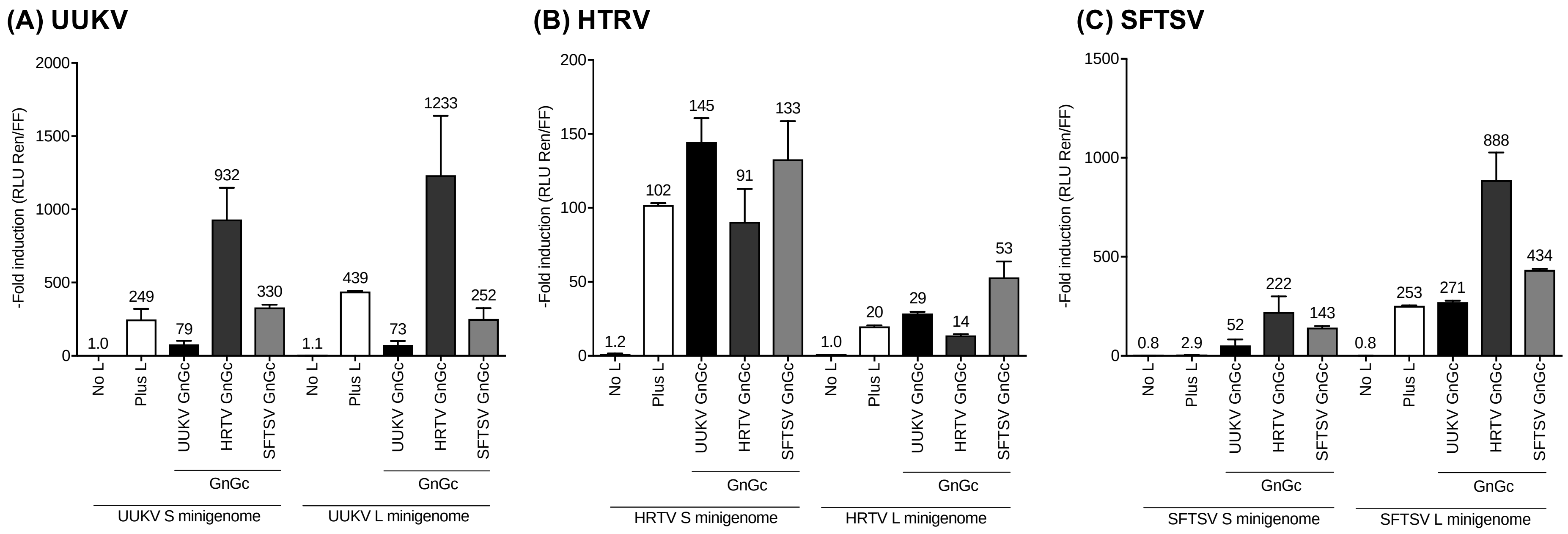

Fig S2. Activity in donor cells used to generate virus-like particles containing S or L segment minigenomes using UUKV, HRTV or SFTSV glycoproteins. VLPs containing UUKV (A), HRTV (B) or SFTSV (C) S or L segment-based minigenomes were generated in donor HuH7-Lunet-T7 cells, using UUKV, HRTV or SFTSV glycoproteins. Mock controls included VLPs generated lacking L protein (No L) or lacking glycoprotein-coding plasmids (Plus L) in the transfections reactions. 72 h post transfection the *Renilla* and firefly luciferase activities in donor cells were measured. Minigenome activity is expressed as the fold induction of normalised luciferase units relative to the background control (absence of L-expressing plasmid). Bars and the number above represent the mean fold induction ± SD of one representative experiment of two performed in duplicate.

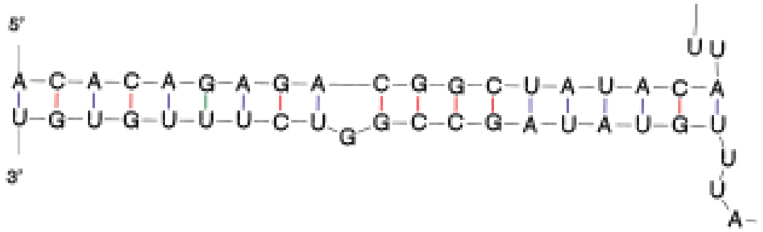

Fig S3. Antigenomic HRTV UTR structure.
As predicted by mFOLD webserver.
